# Towards sequence-based prediction of mutation-induced stability changes in unseen non-homologous proteins

**DOI:** 10.1186/1471-2164-15-S1-S4

**Published:** 2014-01-24

**Authors:** Lukas Folkman, Bela Stantic, Abdul Sattar

**Affiliations:** Institute for Integrated and Intelligent Systems, Griffith University, Brisbane, Australia; NICTA - National ICT Australia, Kragujevac, Australia

**Keywords:** protein mutation, stability changes, machine learning

## Abstract

**Background:**

Reliable prediction of stability changes induced by a single amino acid substitution is an important aspect of computational protein design. Several machine learning methods capable of predicting stability changes from the protein sequence alone have been introduced. Prediction performance of these methods is evaluated on mutations unseen during training. Nevertheless, different mutations of the same protein, and even the same residue, as encountered during training are commonly used for evaluation. We argue that a faithful evaluation can be achieved only when a method is tested on previously unseen proteins with low sequence similarity to the training set.

**Results:**

We provided experimental evidence of the limitations of the evaluation commonly used for assessing the prediction performance. Furthermore, we demonstrated that the prediction of stability changes in previously unseen non-homologous proteins is a challenging task for currently available methods. To improve the prediction performance of our previously proposed method, we identified features which led to over-fitting and further extended the model with new features. The new method employs *Evolutionary And Structural Encodings with Amino Acid parameters* (EASE-AA). Evaluated with an independent test set of more than 600 mutations, EASE-AA yielded a Matthews correlation coefficient of 0.36 and was able to classify correctly 66% of the stabilising and 74% of the destabilising mutations. For real-value prediction, EASE-AA achieved the correlation of predicted and experimentally measured stability changes of 0.51.

**Conclusions:**

Commonly adopted evaluation with mutations in the same protein, and even the same residue, randomly divided between the training and test sets lead to an overestimation of prediction performance. Therefore, stability changes prediction methods should be evaluated only on mutations in previously unseen non-homologous proteins. Under such an evaluation, EASE-AA predicts stability changes more reliably than currently available methods.

**Electronic supplementary material:**

The online version of this article (doi:10.1186/1471-2164-15-S1-S4) contains supplementary material, which is available to authorized users.

## Background

Even a single amino acid substitution, a mutation, in a protein sequence may result in significant changes in protein stability, structure, and therefore in protein function as well [1]. Hence, accurate prediction of stability changes in protein variants is a crucially important task in computational protein design. Moreover, the ability to predict stability changes may help us understand the relationship between protein mutations and inherited diseases.

As more experimental data about stability changes became available in the ProTherm database [2], machine learning methods for predicting stability changes emerged. Broadly, they can be categorised as *structure-based* and *sequence-based* methods. *Structure-based* methods [3–8] require protein three-dimensional structure on the input which can be limiting if the experimentally solved structure is not available. Thus, with the immense amounts of data coming from the genome sequencing projects, the *sequence-based* methods are valuable tools for studying protein variants. In this work, we focused our attention on the *sequence-based* methods.

Traditionally, sequence-based methods make their predictions based on the amino acid identities of the mutation site and several neighbouring residues [9–12]. Alternatively, the mutation site and its neighbouring residues can be encoded with a set of amino acid properties to account for physicochemical differences among amino acids [13, 14]. In our recent work [15], we proposed a method that combines amino acid identities of the mutation site neighbourhood with evolutionary and predicted structural features.

All of the studies referenced above were able to report a high cross-validation accuracy between 77% and 86% (Matthews correlation coefficient between 0.39 and 0.65) when classifying mutations as stabilising or destabilising [9–15]. Regarding the real-value prediction of stability changes, the correlation of the predicted and experimentally measured stability changes reached a correlation coefficient of 0.62 to 0.83 [9–11, 15]. Nevertheless, an assessment study [16] indicated that the prediction performance of these methods on an independent test set is considerably lower than stated in the original studies.

There might be several aspects to why currently available methods did not perform well in the independent assessment. For example, as shown in [10], when the data set used for training and evaluation did not contain multiple records for measurements of the same mutation at different experimental conditions, sensitivity (accuracy on positive examples) of the proposed method decreased from 71% to 28%. When the evaluation was further restricted to only proteins with low sequence similarity to the training set, sensitivity reached only 15%. These findings [10, 16] suggest that currently available methods may suffer from over-fitting on the mutations and proteins that they experienced during training. However, the over-fitting problem is not apparent from the performance results reported in the original studies. This may mean that the evaluation scheme needs to be revisited.

Commonly, stability changes prediction performance is evaluated using cross-validation which randomly divides all data set examples into *k* folds where *k−*1 folds are used for training and one fold for testing. This is repeated *k* times, each time with a different test fold. Typically, a stability changes data set consists of 1,000 to 3,000 examples describing various mutations in up to 90 different proteins. Upon randomly dividing examples of such a data set into *k* folds, different mutations of the same protein, and even the same residue, can be found among several folds. This means that even though a prediction method is tested on *mutations* unseen during training, different mutations of the *same protein*, and even the *same residue*, can be found in both training and test folds. This introduces bias if a method is designed using a data set in which correlation among different mutations of the *same protein*exists. For instance, the data set compiled in this study contains 1,914 unique mutations in 74 different non-homologous proteins (960 different residues). In 68 proteins which have more than one mutation record available, 78% of mutations agree with the prevailing sign of stability changes for the given protein. This number rises to 82% when we analyse mutations in each residue position with more than one mutation record available. Because of this correlation in the available data, stability changes prediction methods should be evaluated solely on mutations in previously *unseen non-homologous proteins*.

In this study, we provided experimental evidence of the limitations of the evaluation commonly used for assessing the prediction performance. Next, we proposed an evaluation scheme that can detect over-fitting on mutations in residues and proteins encountered during training. To achieve this, the evaluation is done solely on previously unseen proteins with sequence similarity below 25%. Finally, to improve the prediction performance of our previously proposed method [15], we identified features which led to over-fitting and further extended the model with new features. The new method bases its predictions on *Evolutionary And Structural Encodings with Amino Acid parameters* (EASE-AA). We compared EASE-AA with currently available methods for both classification and real-value prediction of stability changes. Our results show that EASE-AA increases prediction performance on unseen non-homologous proteins.

## Methods

Stability changes prediction can be viewed as a machine learning classification problem if we are only interested in the direction of the stability change: stabilising (an increase in the free energy of unfolding) or destabilising (a decrease in the free energy of unfolding). If we are concerned with the real-value prediction, it is a regression problem. In this study, we proposed a method referred to as EASE-AA: *Evolutionary And Structural Encodings with Amino Acid parameters*. EASE-AA encompasses two models: one trained for classification and one for regression.

### Predictive features for EASE-AA

For machine learning prediction of stability changes, each mutation needs to be encoded with a number of predictive features. We combined evolutionary and predicted structural features with physical amino acid parameters to design EASE-AA.

#### Evolutionary features

Some residues in a protein sequence are more conserved within the family of related proteins than others. Notably, functionally important sites tend to be conserved. This has been previously exploited for the prediction of deleterious mutations [17–23]. We introduced a range of evolutionary features for the prediction of stability changes in our recent work [15]. There, the best performing model included two evolutionary features: SIFT *score* (*S*) and *mutation likelihood* (*M*).

SIFT [20] predicts whether a mutation affects the function of a protein. It is calculated from a scaled probability matrix of possible amino acid substitutions generated from a multiple sequence alignment of related sequences. SIFT scores range from 0 to 1 where scores below 0.05 are predicted as deleterious mutations. We ran SIFT using the Swiss-Prot and TrEMBL databases with sequences more than 90% identical to the query removed.

The feature *mutation likelihood* (*M*) expresses the probability of the introduced amino acid to appear in the multiple sequence alignment of related proteins. To calculate this feature, three iterations of PSI-BLAST [24] in default configuration were used to search the NCBI non-redundant database. Then, *M* was extracted from the last position specific scoring matrix (PSSM). We divided *M* by 10 for normalisation so that most values fell within the range of *−*1 and 1.

#### Structural features

It has been shown previously that stability changes prediction can be guided by observing structural properties describing the secondary structure and accessible surface area of the mutated residue [25]. However, structural information is not available in the case of *sequence-based* prediction of stability changes. Nevertheless, in our recent work [15], we found that *predicted* structural features can supplement the missing structural information. There, the best performing model included features *secondary structure type* (*SS)* and *accessible surface area* (*ASA*) for classification and real-value prediction, respectively. We included both features in EASE-AA and further extended the model with predicted *disorder probability* (*D*).

We used the multi-step neural network method SPINE-X [26] for the prediction of the secondary structure type and accessible surface area of each mutation site. For the prediction of the disorder probabilities, the neural network method SPINE-D [27] was used. Since feature *SS* describes the mutation site as either *α*-helix, *β*-sheet, or coil, it was represented in three binary inputs (1 was used to determine the secondary structure type present, 0 otherwise). Unlike in our previous work where feature *ASA* encoded mutation site as buried or exposed, we included the real value of the predicted accessible surface area in EASE-AA.

#### Amino acid parameters

Different sets of physical parameters for encoding the substituted and neighbouring amino acids have been introduced for the prediction of stability changes [4, 5, 13, 14]. Recently, calculating the difference in physical parameters between the introduced and deleted amino acids was proposed [8]. We followed this methodology and applied it to seven representative parameters including hydrophobicity, volume, polarisability, isoelectric point, helix probability, sheet probability, and a steric parameter (graph shape index). These parameters were first introduced in [28] and later applied to the prediction of secondary structure [26]. We used the scaled values of the seven parameters from [29]. We refer to the predictive feature encompassing the differences of seven physical parameters for the introduced and deleted amino acids as *amino acid parameters* (*AAP)*.

#### Final set of predictive features

The final set of predictive features for EASE-AA was composed of the following features: *S* (1 real-value input), *M* (1 real-value input), *SS* (3 binary inputs), *ASA* (1 real-value input), *D* (1 real-value input), *AAP* (7 real-value inputs). Compared to our previous work [15], EASE-AA extends the predictive model with the disorder probability (*D)* and seven amino acid parameters (*AAP)*. Moreover, we excluded 6*×*20 binary inputs describing the three and three amino acid neighbours to the left and right from the mutation site. Also, EASE-AA does not include 20 inputs encoding the identities of the deleted and introduced amino acids. This approach resulted in an overall reduction of the number of input attributes from 145 to only 14. Hence, EASE-AA is presumably more robust against over-fitting.

### Support vector machines

Support vector machines (SVM) [30] are machine learning algorithms which can approximate non-linear functions by mapping the inputs to a high-dimensional feature space using a kernel function and then, solving a linear problem by finding a maximum margin separating hyperplane. We adopted the radial basis kernel function because it has been shown to perform well for predicting stability changes [10]. To implement our method with SVM, we used the LIBSVM library [31].

The regularisation parameter *C* and the radial basis kernel width parameter *γ* need to be chosen to optimise SVM performance. In the case of real-value prediction, another parameter (ε), determining the error neglected during training, is required. For classification, a parameter setting the weight (*w)* of the penalty for training error on positive examples should be set if the number of positive and negative examples in the data set is unbalanced. For each experiment, we optimised these parameters by running a *grid search* using 10-fold cross-validation on the training set so that the highest Matthews correlation coefficient (MCC) and lowest root mean square error (RMSE) were reached for classification and real-value prediction, respectively. In the grid search, we considered all possible combinations of *C* ∈ {2^*−*5^, 2^*−*3^, . . . , 2^15^}, *γ* ∈ {2^*−*15^, 2^*−*13^, . . . , 2^1^}, and *w* ∈ {1, 1.5, 2, 2.5, 3} for classification, and *C* ∈ {2^*−*1^, 2^0^, . . . , 2^6^}, *γ* ∈ {2^*−*15^, 2^*−*14^, . . . , 2^0^}, and *ε* ∈ {2^*−*8^, 2^*−*7^, . . . , 2^*−*1^} for real-value prediction. The range values for *C, γ*, and *ε* were taken from the LIBSVM grid search [31] and extended to suit all methods assessed in this study. We also considered using a data-driven approach for optimising the kernel width parameter (*γ*) [32], however, for the relatively small size of our data set, the grid search was a sufficient solution.

As mentioned above, we decided to optimise the SVM performance in terms of MCC in the case of classification. MCC is a measure of prediction performance that provides more relevant information than classification accuracy in cases when the data set is severely biased against one class of examples. Since destabilising (negative) mutations prevail in the available experimental data (74% in our data set), optimising on MCC allowed us to achieve a more balanced performance in terms of correctly predicted both stabilising and destabilising mutations.

### Data sets

We compiled a data set of free energy stability changes from the ProTherm database [2] (February 2013). There, a stability change is defined as the difference in the unfolding free energy: ΔΔ*G*_*u*_[kcal mol^*−*1^] = Δ*G*_*u*_(*mutant*) *−* Δ*G*_*u*_(*wild*-*type*). Hence, for the classification problem, we defined stabilising mutations (ΔΔ*G*_*u*_*≥* 0) to be the positive examples and destabilising mutations (ΔΔ*G*_*u*_*<*0) to be the negative examples.

We extracted 3,329 mutations with listed stability changes and cross-checked all the sources where the measurements came from. We found that incorrect values (mostly the sign of ΔΔ*G*_*u*_) had been entered from at least 18 sources. We corrected stability changes for all relevant (*>*230) mutations in the extracted data set. Next, we removed all duplicate entries of the same amino acid substitutions (different concentrations of chemicals, stability changes of the protein intermediate state, etc.). If several measurements of the same mutation under the *same* experimental conditions were present, we averaged the stability changes and kept only a single entry. If several measurements of the same mutation under *different* experimental conditions were present, we kept only the measurement closest to the physiological pH 7. We removed the other entries because we believe that there is not enough data to appropriately model stability changes of the same mutation under different experimental conditions. Moreover, stability changes of mutations differing only in temperature and pH were highly correlated in the extracted data set.

Finally, we identified 74 clusters of homologous sequences with more than 25% sequence similarity using BLASTCLUST [33]. If there were several measurements of the same amino acid substitution within a single cluster, we kept only the measurement closest to the physiological pH 7. This process yielded a non-redundant data set of 1,914 mutations in 95 different proteins grouped into 74 non-homologous clusters. We refer to this data set as S1914. The data set is available in Additional file 1.

### Experiments and different evaluation schemes

Three different evaluation schemes were compared in this study: *unseen-mutation, unseen-residue*, and *unseen-protein* evaluation. The most commonly used evaluation of sequence-based stability changes prediction methods is on unseen mutations. There, mutations are *randomly* divided into training and test sets (or into cross-validation folds). This means that different mutations in the same protein, and even in the same residue, can be used for training and testing. Because of the correlation in the available data sets, the most important drawback of the *unseen-mutation* evaluation is that even methods which over-fit on residue positions and proteins from the training set can achieve high prediction performance on the test set (or in cross-validation).

The *unseen-residue* evaluation guarantees that all mutations in the same residue position of a protein (or its homologue) exist either in the training or the test set (or in distinct folds for cross-validation). Hence, methods which over-fit on mutations in residue positions from the training set are unlikely to achieve good prediction performance on the test set (or in cross-validation). The *unseen-residue* evaluation has been previously adopted for the design of a three-state prediction method I-Mutant3.0 [34].

Finally, the strictest assessment we considered was the *unseen-protein* evaluation. In this case, all mutations in the same protein and its homologues were used exclusively for either training or testing. Thus, if a prediction method cannot generalise well for mutations in previously unseen non-homologous proteins, it is unlikely to achieve a good performance under this evaluation.

#### *Training set, test set, and cross-validation folds*

To achieve an unbiased evaluation, we split the S1914 data set randomly into training and independent test sets with a ratio of 2 : 1. We repeated this process 10 times producing 10 different training/test splits. Each training set was further divided into 10 cross-validation folds. The ratio of positive and negative examples in the 10 folds and in the independent test set was kept close to that of the original data set. Cross-validation using the 10 folds was employed to optimise the performance of the evaluated methods. Then, each method was trained on the whole training set and tested on the examples in the independent test set. The whole process was repeated 10 times, utilising the 10 different training/test splits. Finally, the results of the 10 experiments were averaged.

We compared *unseen-mutation, unseen-residue*, and *unseen-protein* evaluation schemes in this study. Hence, splitting into the training and independent test sets as well as to the cross-validation folds was executed according to one of these three evaluation schemes for different experiments.

#### Comparison with currently available methods

We compared the prediction performance of our new method (EASE-AA) with our previously proposed method [15] which also employs evolutionary and structural encodings (thus, we refer to it as EASE). To further show how prediction performance varies when different evaluation schemes are employed, we evaluated another two sequence-based methods: I-Mutant2.0 [9] and MUpro [10]. These two methods had also been included in an independent assessment study [16]. We did not compare with I-Mutant3.0 [34] because it predicts stability changes into three states (stabilising, destabilising, and neutral).

To be able to asses I-Mutant2.0 and MUpro under different evaluation schemes, we implemented the two methods according to their description in the original publications. Therefore, rather than performing a comparison with the actual methods, we performed a comparison with the set of predictive features proposed for I-Mutant2.0 and MUpro. This approach allowed us to achieve a fair comparison of all four methods by optimising the SVM parameters and re-training the SVM models for every experiment on the same training set.

I-Mutant2.0 bases its prediction on the occurrence frequencies of the sequential neighbourhood, hence, we refer to our implementation as SEQ-FREQ. MUpro uses amino acid identities of neighbouring residues, thus, we refer to our implementation of this method as SEQ-NEIGHB.

### Evaluation measures

The prediction performance in the classification task was assessed in terms of Matthews correlation coefficient (MCC), classification accuracy (Q_2_), sensitivity (Se), specificity (Sp), positive predictive value (PPV), and negative predictive value (NPV):123456

where *TP, TN, FP*, and *FN* refer to the number of true positives, true negatives, false positives, and false negatives, respectively. Furthermore, we assessed the classification performance by plotting the receiver operating characteristic (ROC) curve and calculating the area under the ROC curve (AUC). A ROC curve plots the true positive rate (sensitivity) as a function of the false positive rate (100 *−* specificity) at different prediction thresholds.

For real-value prediction, performance was assessed in terms of Pearson correlation coefficient (*r)* and root mean square error (RMSE):78

## Results

We compared the prediction performance of the two methods from the literature, I-Mutant2.0 [9] and MUpro [10] (we refer to our implementations of these methods as SEQ-FREQ and SEQ-NEIGHB, respectively), our previously proposed method [15] (denoted as EASE here), and the method designed in this study (EASE-AA). We evaluated both classification and real-value prediction employing the S1914 data set. To achieve a fair comparison of the four methods, each method was re-trained and had the SVM parameters optimised (utilising a cross-validation on the training set) for every experiment.

### Comparison of different evaluation schemes

Commonly, stability changes prediction methods are evaluated using a cross-validation where different mutations of the same protein can be randomly distributed across different folds. We believe that this approach leads to a considerable overestimation of the prediction performance for proteins with low sequence similarity to the training set. To illustrate this in an experiment, we divided our data set into training and independent test sets in three different ways following the *unseen-mutation, unseen-residue*, and *unseen-protein* evaluation schemes (Methods). In the *unseen-mutation* evaluation, different mutations are randomly distributed between the training and test sets, whereas the *unseen-residue* (*unseen-protein*) evaluation guarantees that all mutations in the same residue position (same protein) exist either in the training or the test set. Also, we performed a 10-fold cross-validation on the training set for each training/test split. In this case, the 10 folds were created by *randomly* dividing all mutations. This means that the cross-validation was performed in an *unseen-mutation* evaluation fashion regardless of the evaluation scheme used for the independent test.

Table 1 compares the cross-validation and independent test classification performance of the four assessed methods using the three different evaluation schemes. In cross-validation, EASE yielded the highest Matthews correlation coefficient (MCC) of 0.45. EASE-AA achieved an MCC of 0.43, while it was 0.41 and 0.33 for SEQ-NEIGHB and SEQ-FREQ, respectively. The area under the ROC curve (AUC) ranged from 0.75 to 0.81 for the four methods.

**Table 1:** Comparison of the four methods using the three different evaluation schemes for classification

Method	Evaluation	MCC	Q_2_	Se	Sp	PPV	NPV	AUC
SEQ-NEIGHB	*cross-validation* ^∗^	0.41	77.65	55.09	85.57	57.35	84.45	0.78
	*unseen-mutation*	0.36	75.80	51.16	84.48	53.74	83.08	0.78
	*unseen-residue*	0.14	71.65	21.28	89.42	41.52	76.30	0.67
	*unseen-protein*	0.14	69.09	29.45	83.13	38.21	76.89	0.67
SEQ-FREQ	*cross-validation* ^∗^	0.33	72.63	57.37	77.98	48.00	83.92	0.75
	*unseen-mutation*	0.29	69.27	58.64	73.02	43.36	83.36	0.74
	*unseen-residue*	0.22	69.30	43.40	78.44	41.54	79.70	0.68
	*unseen-protein*	0.18	65.71	47.70	72.09	37.71	79.55	0.66
EASE	*cross-validation* ^∗^	0.45	78.54	60.95	84.72	58.33	86.08	0.81
	*unseen-mutation*	0.41	77.05	57.91	83.80	55.74	84.96	0.81
	*unseen-residue*	0.26	73.26	37.63	85.84	48.40	79.59	0.72
	*unseen-protein*	0.23	71.27	39.76	82.44	44.50	79.44	0.70
EASE-AA	*cross-validation* ^∗^	0.43	76.69	63.35	81.37	54.43	86.35	0.80
	*unseen-mutation*	0.38	74.71	60.64	79.66	51.23	85.17	0.79
	*unseen-residue*	0.34	73.23	56.11	79.27	48.86	83.65	0.76
	*unseen-protein*	0.35	73.24	58.79	78.36	49.04	84.30	0.77

^∗^ cross-validation folds were created by dividing mutations randomly (*unseen-mutation* cross-validation)

For the independent test, we used three different evaluation schemes: *unseen-mutation, unseen-residue*, and *unseen-protein*. The *unseen-mutation* evaluation resulted only in a marginally lower performance compared to the cross-validation results (an MCC and AUC decrease of up to 0.05 and 0.01, respectively). However, if the *unseen-residue* or *unseen-protein* evaluation was employed, the performance of all four methods decreased considerably when compared to the cross-validation results. The largest decline was for SEQ-NEIGHB. In this case, the MCC decreased by 0.27 (from 0.41 to 0.14) for both *unseen-residue* and *unseen-protein* evaluations. Our new method (EASE-AA) experienced the smallest decrease in prediction performance. EASE-AA's MCC declined by 0.09 and 0.08 (from 0.43 to 0.34 and 0.35) for predictions on unseen residues and unseen proteins, respectively.

**Figure 1:**
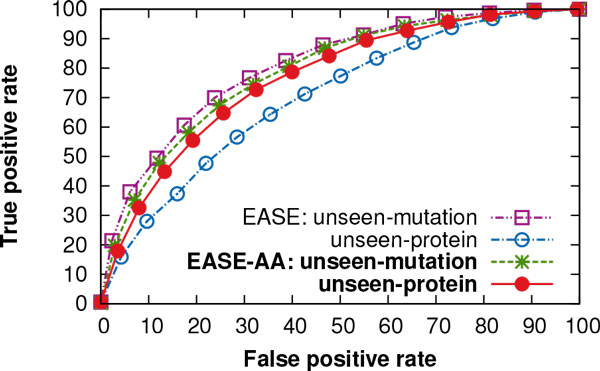
**ROC curves performance of EASE and EASE-aa using two different evaluation schemes**. The true positive rate of EASE and EASE-AA is shown as a function of the false positive rate at different prediction thresholds. These are independent test results using the *unseen-mutation* and *unseen-protein* evaluation. While for EASE-AA the area under the ROC curve (AUC) declined only by 0.02 when comparing the *unseen-mutation* and *unseen-protein* evaluation, EASE yielded a decrease of 0.11.

The receiver operating characteristic (ROC) curves in Figure 1 compare the true positive rate of EASE and EASE-AA as a function of the false positive rate for the *unseen-mutation* and *unseen-protein* evaluation. We were interested in studying the decrease in the independent test performance between the two evaluation schemes. While in the case of EASE-AA, the area under the ROC curve (AUC) declined only by 0.02 for the *unseen-protein* evaluation, EASE yielded an AUC decrease of 0.11. The ROC curves of EASE and EASE-AA for the *unseen-residue* evaluation were similar to those for the *unseen-protein* evaluation (not shown in the figure).

The results from the real-value prediction experiment showed the same trend in the relative comparison of the four methods under the three different evaluation schemes (Table 2). Prediction performance decreased when comparing the results from the *unseen-mutation* with the *unseen-residue* or *unseen-protein* evaluation. The smallest decrease in prediction performance was yielded by EASE-AA. Also, EASE-AA was the best performing method in predicting real-value stability changes in previously unseen residues and unseen proteins.

**Table 2:** Comparison of the four methods using the three different evaluation schemes for real-value prediction

Method	Evaluation	r	RMSE
SEQ-NEIGHB	*cross-validation* ^∗^	0.63	1.38
	*unseen-mutation*	0.59	1.46
	*unseen-residue*	0.37	1.60
	*unseen-protein*	0.34	1.62
SEQ-FREQ	*cross-validation* ^∗^	0.56	1.47
	*unseen-mutation*	0.53	1.53
	*unseen-residue*	0.40	1.60
	*unseen-protein*	0.33	1.67
EASE	*cross-validation* ^∗^	0.68	1.30
	*unseen-mutation*	0.64	1.38
	*unseen-residue*	0.44	1.55
	*unseen-protein*	0.40	1.60
EASE-AA	*cross-validation* ^∗^	0.58	1.44
	*unseen-mutation*	0.55	1.50
	*unseen-residue*	0.53	1.46
	*unseen-protein*	0.50	1.50

^*^ cross-validation folds were created by dividing mutations randomly (*unseen-mutation* cross-validation)

### Training and evaluation on previously unseen non-homologous proteins

We discovered that the *unseen-mutation* evaluation leads to overestimating the prediction performance for previously unseen residues as well as for previously unseen proteins (Tables 1 and 2). Interestingly, the prediction performance on unseen residues was similar to that on unseen proteins. Therefore, we employed the *unseen-protein* evaluation to further analyse the prediction performance of the four methods.

One of the reasons for the suboptimal performance in predicting unseen proteins could be that we optimised the four methods employing the *unseen-mutation* cross-validation (different mutations of the same protein can appear in different folds). To optimise the compared methods more appropriately to predict stability changes in unseen proteins, we split the training set into 10 folds so that none of the folds shared homologous sequences (*unseen-protein* cross-validation).

Table 3 summarises the cross-validation and independent test results from the classification experiment employing the *unseen-protein* evaluation. For cross-validation, the highest Matthews correlation coefficient (MCC) of 0.37 was achieved by our new method (EASE-AA). This result represents a relative improvement of 48% (an absolute improvement of 0.12) to the second best method (SEQ-FREQ). When we evaluated the four methods on the independent test set, the prediction performance decreased for all methods only marginally. EASE-AA, the best performing method, reached an MCC of 0.36 with a relative improvement of 50% (an absolute improvement of 0.12) compared to the second best method (SEQ-FREQ).

**Table 3:** Classification performance of the four methods optimised for the unseen-protein evaluation

Method	Evaluation	MCC	Q_2_	Se	Sp	PPV	NPV	AUC
SEQ-NEIGHB		0.18	65.84	46.67	72.55	37.34	79.52	0.64
SEQ-FREQ	*unseen-protein*	0.25	63.33	65.71	62.50	38.05	83.87	0.69
EASE	*cross-validation*	0.24	70.99	42.40	81.01	43.91	80.05	0.68
EASE-AA		0.37	72.72	65.35	75.31	48.12	86.11	0.76
SEQ-NEIGHB		0.16	64.77	45.64	71.55	36.24	78.79	0.65
SEQ-FREQ	*unseen-protein*	0.24	61.95	66.97	60.18	37.33	83.72	0.70
EASE	*independent test*	0.22	69.28	43.58	78.38	41.66	79.68	0.69
EASE-AA		0.36	71.53	65.76	73.57	46.85	85.85	0.78

Positive (negative) predictive value (PPV, NPV) refers to the proportion of mutations predicted as stabilising (destabilising) that are truly stabilising (destabilising). EASE-AA yielded PPV and NPV of 46.85% and 85.85%, respectively. These results represent absolute improvements of 9.52 and 2.13 percentage points when compared to SEQ-FREQ. The respective improvements compared to EASE were 5.19 and 6.17 percentage points.

**Figure 2:**
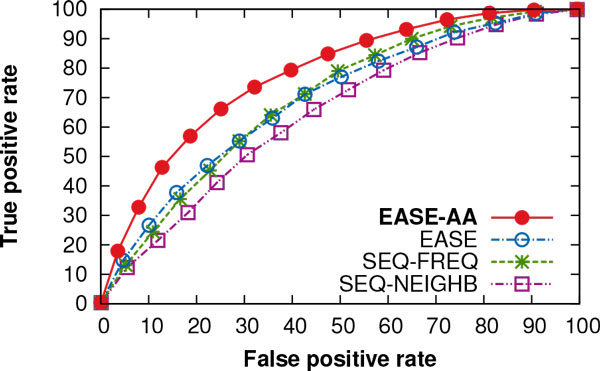
**ROC curves performance of the four methods using the** ***unseen-protein*** **evaluation**. The true positive rate of the four methods is shown as a function of the false positive rate at different prediction thresholds. These are *unseen-protein* independent test results. EASE-AA, EASE, SEQ-FREQ, and SEQ-NEIGHB yielded the area under the ROC curve (AUC) of 0.78, 0.69, 0.70, and 0.65, respectively.

The ROC curves in Figure 2 compare the true positive rate of the four methods as a function of the false positive rate at different prediction thresholds. The figure demonstrates the benefit in terms of the number of correctly predicted positive examples upon employing our method (EASE-AA). EASE-AA achieved an AUC of 0.78, while EASE, SEQ-FREQ, and SEQ-NEIGHB yielded an AUC of 0.69, 0.70, and 0.65, respectively.

We estimated the statistical significance of EASE-AA's improvements in the MCC and AUC over the 10 replications of independent testing using a student *t*-test. The null hypothesis stated that there was no statistical difference in the MCC (AUC) for EASE-AA and each of the three compared methods. The *p*-values associated with this null hypothesis were all less than 0.0005.

The results from the real-value prediction experiment employing the *unseen-protein* evaluation are summarised in Table 4. As in the case of classification, EASE-AA performed the best yielding a correlation coefficient (*r)* of 0.51 and root mean square error (RMSE) of 1.48. These results represent relative improvements of 24% for *r* (an absolute improvement of 0.10) and 5% for RMSE (an absolute improvement of 0.08) to the second method (EASE).

**Table 4:** Real-value prediction performance of the four methods optimised for the unseen-protein evaluation

Method	Evaluation	r	RMSE
SEQ-NEIGHB		0.35	1.67
SEQ-FREQ	*unseen-protein*	0.36	1.67
EASE	*cross-validation*	0.42	1.62
EASE-AA		0.51	1.54
SEQ-NEIGHB		0.34	1.61
SEQ-FREQ	*unseen-protein*	0.36	1.60
EASE	*independent test*	0.41	1.56
EASE-AA		0.51	1.48

Comparing the results when the *unseen-mutation* cross-validation (Tables 1 and 2) and the *unseen-protein* cross-validation (Tables 3 and 4) were used for model optimisation, there does not seem to be a considerable difference in the *unseen-protein* independent test performance. The only exception was SEQ-FREQ which seemed to benefit from the appropriate model optimisation. SEQ-FREQ'S correlation coefficients increased by 0.06 (MCC) and 0.03 (*r)* for classification and real-value prediction, respectively.

### Prediction performance for different types of mutations

EASE-AA outperformed the other three methods (EASE, SEQ-FREQ, and SEQ-NEIGHB) in predicting stability changes in unseen proteins. We were interested in how this improvement varied for different types of mutations. We investigated how accurate (in terms of MCC) each of the four compared methods was in predicting mutations in residues of different secondary structure types (*α*-helix, *β*-sheet, and coil) and accessible surface area assignments (exposed and buried). Residues were defined as exposed if at least 25% of their surface was accessible to the solvent and as buried otherwise. Furthermore, we explored the accuracy of predicting mutations inducing 'small' (ΔΔ*G*_*u*_ ∈ [*−*1, 1]) and 'large' (*|*ΔΔ*G*_*u*_*| >*1 kcal mol^*−*1^) stability changes.

**Figure 3:**
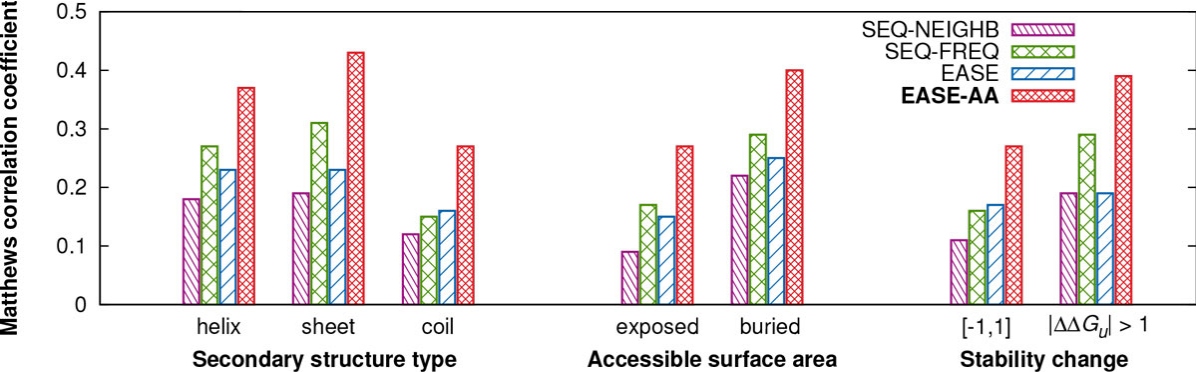
**Prediction performance of the four methods for different types of mutations**. Matthews correlation coefficient (MCC) of SEQ-NEIGHB, SEQ-FREQ, EASE, and EASE-AA is shown as a function of the secondary structure type of the mutated residue, accessible surface area of the mutated residue (threshold of 25% for an exposed residue), and magnitude of the stability change. These are *unseen-protein* independent test results.

Figure 3 shows the Matthews correlation coefficient (MCC) of the four compared methods as a function of the different types of mutations that we investigated. Regarding different secondary structure types, EASE-AA achieved an MCC of 0.37, 0.43, 0.27 for the helical, sheet, and coil residues, respectively. The largest relative improvement to the second best method (SEQ-FREQ) of 80% (an absolute improvement of 0.12) was achieved for coil residues. Interestingly, coil residues were the most difficult to predict for all four methods. For helical and sheet residues, our new method yielded relative improvements of 37% and 39%, respectively (absolute improvements of 0.10 and 0.12). All four methods were able to predict buried mutations more reliably than the exposed ones. The MCC values achieved by EASE-AA for the exposed and buried residues were 0.27 and 0.40, respectively. The respective relative (absolute) improvements to the second best method (SEQ-FREQ) were 59% (0.10) and 38% (0.11). Regarding the performance for mutations with different magnitudes of stability changes, all methods yielded a better performance for mutations causing 'large' stability changes. For this category, EASE-AA achieved an MCC of 0.39, while it was 0.27 for the category of 'small' stability changes. The relative (absolute) improvements for the 'small' and 'large' categories were 69% (0.11) and 34% (0.10), respectively.

Overall, EASE-AA achieved improvements in every category included in the comparison. Moreover, since the absolute improvements were quite balanced among the different types of mutations (ranging from 0.10 to 0.12), EASE-AA yielded higher relative improvements for mutation types which appeared to be more difficult to predict for all of the four compared methods (coils, exposed residues, and 'small' stability changes).

### Predictive features and the improvements yielded by EASE-AA

**Figure 4:**
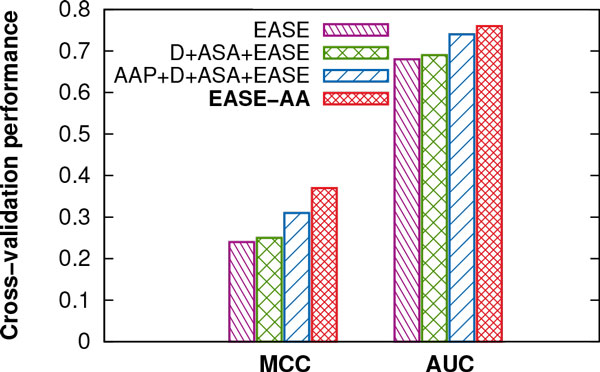
**Performance of different variations of our method during the design of EASE-aa**. The *unseen-protein* cross-validation performance [Matthews correlation coefficient (MCC) and the area under the ROC curve (AUC)] is shown for four different variations of our method. The difference between AAP+D+ASA+EASE and EASE-AA is the removal of the 140 input attributes defining the identities of the deleted, introduced, and neighbouring amino acids.

We found that EASE-AA consistently outperformed our previous work (EASE) when predicting mutations in unseen proteins. Hence, we were interested in how each design step of EASE-AA contributed towards the final improvement. Figure 4 compares the cross-validation performance on the training set in terms of Matthews correlation coefficient (MCC) and the area under the ROC curve (AUC) for EASE, EASE-AA, and two 'intermediate' methods (D+ASA+EASE AND AAP+D+ASA+EASE).

First, we extended EASE with two predicted structural features, *accessible surface area* (*ASA*) and *disorder probability* (*D)*. However, the improvement in the cross-validation performance was only marginal. Next, the seven physical *amino acid parameters* (*AAP)* were added. The inclusion of *AAP* yielded a relative improvement of 24% (an absolute improvement 0.06) in terms of MCC. Finally, we suspected that the 140 input attributes encoding the deleted, introduced, and neighbouring amino acids implemented in EASE may have been leading to over-fitting on residue positions encountered during training. After excluding these 140 inputs (EASE-AA), there was a relative improvement of 19% (an absolute improvement of 0.06) in terms of MCC.

**Figure 5:**
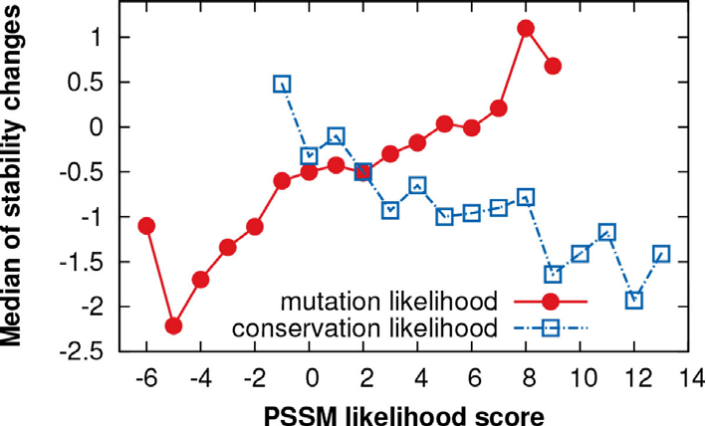
**Relationship between evolutionary conservation and stability changes**. The *median* of experimentally measured stability changes in the S1914 data set is shown as a function of the PSSM scores defining *mutation* and *conservation likehood*. The plot reveals that there is a higher number of destabilising mutations when the mutation likelihood is low and residue conservation high, while stabilising mutations tend to prevail for substitutions which are common in the family of related proteins.

It has been shown previously that structural features [25] and amino acid parameters [13] can be used for the prediction of stability changes. To our best knowledge, evolutionary features have been used only in our previous work [15]. Therefore, we studied the relationship between the evolutionary information and experimentally measured stability changes. We plotted the *median* of stability changes in the S1914 data set as a function of the PSSM scores for the *mutation likelihood* (the same as feature *M)* and *conservation likehood* (*C)* (Figure 5). This plot reveals that as the *median* of stability changes increases, so does the value of *M*, whereas the value of *C* decreases. Hence, the relationship shown in Figure 5 demonstrates that there is a higher number of destabilising mutations when the mutation likelihood is low and residue conservation high. On the contrary, stabilising mutations tend to prevail for mutations which are common in the family of related proteins.

## Discussion

Our main interest was to asses the prediction of stability changes in previously unseen non-homologous proteins. We found that while high prediction performance can be achieved when different mutations of the same protein and residue positions are randomly divided for training and evaluation, it is challenging to predict stability changes in previously unseen proteins. Therefore, our results provide experimental evidence that the commonly adopted *unseen-mutation* evaluation lead to an over-estimation of the prediction performance. To address the prediction of stability changes in unseen proteins, we extended our previous work [15] and proposed a new method (EASE-AA) which was able to outperform the other three methods in our comparison (Figure 2). For classification, EASE-AA achieved a Matthews correlation coefficient (MCC) of 0.36 (Table 3). For real-value prediction, Pearson correlation coefficient (*r)* reached the value of 0.51 (Table 4). Although such a performance may seem relatively low, these results represent relative improvements to the second best method of 50% (MCC) and 24% (*r)*. We believe that one of the limiting factors in yielding more reliable predictions is the scarcity of stabilising mutations and distinct non-homologous proteins available for training. Moreover, as noted elsewhere [5], the variety of available experimental data is quite unbalanced (for instance, 26% of amino acid substitutions were to alanine in our data set).

Comparing the three different evaluation schemes, all four methods achieved a considerably higher prediction performance when the *unseen-mutation* evaluation was used (Table 1). This could be attributed to the correlation that exists among different mutations of the same residue in the available experimental data. Because this correlation cannot be exploited when evaluation is done solely on residues unseen during training, prediction performance of all four methods decreased considerably upon employing the *unseen-residue* evaluation. The *unseen-protein* evaluation further guarantees that all mutations of the same protein are used either for training or evaluation. Performance of all four methods changed only marginally when comparing the results from the *unseen-residue* and *unseen-protein* evaluation. This is most likely because of the absence of 'protein-wide' predictive features in the four compared methods. Hence, the *unseen-residue* evaluation was just as challenging as the one on unseen proteins.

When comparing performance of EASE-AA with our previously proposed method [15], the reasons for the improvements are twofold. Firstly, we excluded features encoding the identities of the deleted, introduced, and neighbouring amino acids because they led to over-fitting on residues and proteins encountered during training (Figure 4). Secondly, we incorporated the differences in seven representative physical parameters for the deleted and introduced amino acids (feature *AAP)*. For instance, the difference in the physical parameter encoding the volume of an amino acid can suggest if the mutation may induce strain in the protein structure due to the large size of the introduced residue. Similarly, a change in the hydrophobicity can suggest an introduction of disturbing interactions in the hydrophobic core of the protein.

Our new method adopts the evolutionary predictive features proposed in our previous work [15]. Actually, the observation that functionally important sites tend to be evolutionary conserved has been previously exploited for the prediction of deleterious mutations [20]. However, there are other reasons than the location of functional sites for the existence of conserved regions. For example, conserved regions play an important part in stabilising the structure of a protein [35]. We demonstrated that the relationship between evolutionary predictive features derived from PSSM and experimentally measured stability changes from our data set agree with these general assumptions about sequence conservation (Figure 5).

It seems that the most difficult mutations to predict are either located in coil and exposed residues or those which cause only small stability changes (within the range of *−*1 and 1 kcal mol^*−*1^). Prediction performance of all four methods in these three categories was lower than for any other category of different types of mutations that we investigated (Figure 3). These findings are in agreement with the results reported in a study about a neural network structure-based method [3]. Also, it has been shown previously that different interactions govern stability changes in exposed and buried residues [36]. Regarding the prediction of 'small' stability changes, naturally, it is harder to differentiate among subtle changes. Moreover, experimental data is affected by the error of measurement which can be as large as *±*0.48 kcal mol^*−*1^[37]. Hence, the strict classification of the 'small' stability changes as stabilising or destabilising can be misleading [34, 13].

Overall, our new method, EASE-AA, achieved improvements in all categories of different types of mutations that we investigated. Moreover, EASE-AA yielded higher relative improvements for the types of mutations which were the most challenging to predict for all four compared methods. These results demonstrate the robustness of the performance of our new method in predicting stability changes in previously unseen non-homologous proteins.

## Conclusions

In this work, we demonstrated how performance varies depending on the evaluation scheme employed. This is most likely because the machine learning methods are prone to over-fitting on mutations in residues and proteins encountered during training. When the evaluation on previously *unseen non-homologous proteins* was used, currently available methods could not reliably predict stability changes. To address this problem, we designed a new method which is based on *Evolutionary And Structural Encodings with Amino Acid parameters* (EASE-AA). Compared to our previous work [15], features leading to over-fitting were removed and the model was extended with differences in seven physical amino acid parameters.

EASE-AA achieved a Matthews correlation coefficient of 0.36 and was able to classify correctly 66% of the stabilising and 74% of the destabilising mutations. For real-value prediction, EASE-AA achieved a correlation between predicted and experimentally measured stability changes of 0.51. Even though this performance may seem relatively low, EASE-AA predicts stability changes in unseen proteins more accurately than the other three methods in our comparison. This further highlights another important finding of this study that the prediction performance of currently available methods is often overestimated due to randomly dividing different mutations of the same protein, and even the same residue, for training and evaluation.

## Availability of supporting data

The data sets supporting the results of this article are included within the article and its additional files. The source code of our method is available for download from http://www.ict.griffith.edu.au/bioinf/ease.

## Electronic supplementary material

Additional file 1: **Data set S1914**. The files containing the S1914 data set, as well as training, cross-validation, and test splits are available in a white-space-delimited tabular text format. All files are compressed in a single zip archive *S1914.zip*. (ZIP 2 MB)
